# Biological Features, Antimicrobial Susceptibility and Phenotypic Characterization of *Candidozyma auris* CDC B11903 Grown at Different Temperatures

**DOI:** 10.3390/jof11090625

**Published:** 2025-08-26

**Authors:** Terenzio Cosio, Natalia Pedretti, Luca Spaggiari, Luigi Tordelli Ruda, Samyr Kenno, Samuele Sabbatini, Enrico Salvatore Pistoia, Manola Comar, Claudia Monari, Andrea Ardizzoni, Roberta Gaziano, Eva Pericolini

**Affiliations:** 1 Department of Experimental Medicine, University of Rome “Tor Vergata”, 00133 Rome, Italy; terenziocosio@gmail.com (T.C.); pistoiae@uniroma2.it (E.S.P.); 2 Department of Basic Biotechnological Sciences, Intensive and Perioperative Clinics, Università Cattolica del Sacro Cuore, L.go F. Vito 1, 00168 Rome, Italy; 3 Department of Medical Sciences, University of Trieste, 34149 Trieste, Italy; natalia.pedretti@units.it (N.P.); manola.comar@units.it (M.C.); 4 Department of Surgical, Medical, Dental and Morphological Sciences with Interest in Transplant, Oncological and Regenerative Medicine, University of Modena and Reggio Emilia, 41124 Modena, Italy; luca.spaggiari@unimore.it (L.S.); samyr.kenno@unimore.it (S.K.); andrea.ardizzoni@unimore.it (A.A.); 5 Department of Medicine and Surgery, University of Perugia, 06132 Perugia, Italy; luigi.tordelliruda@dottorandi.unipg.it (L.T.R.); samuele.sabbatini@unipg.it (S.S.); claudia.monari@unipg.it (C.M.); 6 Department of Advanced Translational Microbiology, Institute for Maternal and Child Health—IRCCS Burlo Garofolo, 34137 Trieste, Italy

**Keywords:** *Candida*, *Candidozyma auris*, thermo-tolerance, virulence factors, antimicrobial susceptibility

## Abstract

Thermo-tolerance is a virulence factor responsible for the emergence of new fungal pathogens, including *Candidozyma auris* (formerly classified as *Candida auris*, *C. auris*). It has been shown that in *C. auris* the thermo-tolerance, as well as other virulence traits, such as the ability to aggregate, to form pseudo-hyphae, or to produce melanin are strain-specific features. Here, we investigated the impact of different temperatures (25 °C, 37 °C and 42 °C) on the phenotypic and virulence profile of *C. auris* strain CDC B11903. The results show a positive correlation between the resistance to antifungals and increasing temperature from 25 °C to 37 °C, while no differences were observed between 37 °C and 42 °C, except for Anidulafungin. Furthermore, *C. auris* growth was impaired at 25 °C as compared to 37 °C and 42 °C. Except for the haemolytic activity, which increased with rising temperatures, phospholipase, lipase and biofilm production were found at all tested temperatures. Moreover, the ability to produce melanin was observed only at 37 °C and 42 °C. The capacity to grow as pseudo-hyphae or in clusters and to adhere to both biotic and abiotic surfaces were observed at all the temperatures tested, with increased propensity of *C. auris* to adhere to abiotic surfaces with rising temperatures. The results underline the thermo-tolerance of *C. auris* strain B11903 and its increased virulence profile at human body temperature both in physiological (37 °C) and febrile state (42 °C).

## 1. Introduction

*Candida* species are among the most prevalent human fungal pathogens worldwide and represent the fourth most common cause of hospital-acquired bloodstream infections in the US. Notably, *C. albicans*, *C. glabrata*, *C. parapsilosis* and *C. tropicalis* account for approximately 90% of cases of candidemia [1]. Recently, *Candidozyma auris*, a new opportunistic *Candida* species, has emerged and quickly spread worldwide [2]. In only seven years this yeast, which is difficult to treat and displays clonal inter- and intra-hospital transmission, has become widespread across several countries, from the U.S.A to China, passing through Iran and Singapore [3], causing a broad range of healthcare-associated invasive infections, including not only central venous catheter (CVC) correlated candidemia, but also pericarditis, respiratory and urinary tract infections [4]. Invasive infections by *C. auris* mostly occur in critically ill patients, especially patients in intensive care units (ICUs) and those undergoing invasive procedures [5]. This fungus is of clinical concern for several reasons: (1) about 90% of the clinically isolated strains are resistant to fluconazole and some strains are resistant to all currently available antifungal drugs including Amphotericin B and echinocandins, thus dramatically limiting the therapeutic options [6]; (2) although the recently setting up of rapid methods, such as the “Clade-Finder” [7] has been developed to improve the clade determination for *C. auris*, the correct identification in daily routine is still challenging, often leading to inappropriate management; (3) people can be colonized by *C. auris* without being aware of it, and such colonization can persist for long time and cause epidemic outbreaks in healthcare facilities, although the possible spread in communities cannot be excluded; (4) the ability to evade the host immune system makes this fungus highly virulent [8]; (5) it causes a high mortality rate in the invasive forms (in-hospital mortality rate of *C. auris* candidemia ranges from 30 to 60%) [6]; (6) it has the ability to form a biofilm that makes the fungus very resistant to disinfectants and therefore particularly difficult to eliminate from surfaces. *C. auris* strains are classified in six distinct clades named after the geographical areas where the strains were first isolated: South Asian (clade I), East Asian (clade II), African (clade III), South American (clade IV), Iran (clade V) and Singapore (clade VI) [9,10,11]. Differences in genetic backgrounds, biochemical characteristics and antifungal susceptibility patterns make each clade unique [12]. Interestingly, not only antifungal susceptibility pattern seems to be different among the different clades as well as within the same clade, but also its virulence traits, such as thermo-tolerance, halo-tolerance, melanization and the capacity to form pseudo-hyphae seem to be strain-dependent [13,14,15,16,17]. Several aspects of *C. auris* biology, such as its genomic and phenotypic characteristics, as well as its pathogenicity, have been studied [18,19,20]. Interestingly, a possible role exerted by the natural environment on the emergence and transmission of this fungus has been hypothesized. It has been suggested, for over a decade, that mycoses in mammals are infrequent, and this is probably due to the struggle of most fungal species to survive and replicate at the temperatures occurring in mammalian bodies [21]. Recently, it has been reported that some fungal species such as *Cryptococcus* spp., *Coccidioides posadasii* and *Saccharomyces cerevisiae* [22] are able to increase their thermal tolerance, therefore becoming able to adapt to warmer temperatures in the environment, and consequently defeating the protection provided by the endothermy to mammalians [23]. For this reason, global warming is becoming a matter of concern, because it exerts selective pressure on some fungal species that may become pathogenic and lead to the emergence of novel fungal diseases. The quick and simultaneous appearance of *C. auris* in different and distant regions of our planet supports the “global warming emergence hypothesis”, pointing to this species as a prototype of novel fungal pathogens capable of breaking the mammalian endothermal barrier as a consequence of the global warming adaptation [24,25]. Indeed, *C. auris* is characterized by genetic variability, capacity to form biofilm, resistance to antifungal drugs and several mechanisms (such as thermotolerance and halotolerance) that make this species capable of adapting to changes in its environment [26,27]. In addition, variations in temperature, salinity and pH have been demonstrated to play a role in the capacity of *C. auris* to cause disease [28,29,30]. As mentioned above, most of the virulence traits of *C. auris* are strain-specific; hence the specific biological and phenotypical behaviour are peculiar of each single strain and could modify its virulence potential. Here, we studied the biological features, antimicrobial susceptibility and phenotypic characteristics of *C. auris* strain CDC B11903 in comparison to the *C. albicans* reference strain B90028, grown at three different temperatures: 25 °C, 37 °C and 42 °C to assess the impact of temperature on their virulence and phenotypes.

## 2. Materials and Methods

### 2.1. Candida Strains and Growth Conditions

The experiments were carried out using the *C. auris* reference strain CDC B11903 and the *C. albicans* reference strain B90028. Both strains were grown on Sabouraud Dextrose Agar (SDA, Difco Laboratories, Detroit, MI, USA), supplemented with chloramphenicol (SDA + CAF), for 24 h at 30 °C. After incubation, *Candida* yeasts were harvested by washing the slant culture with sterile saline solution (0.85% NaCl; Sigma-Aldrich, Burlington, MA, USA). The cell density of *Candida* suspensions was estimated by direct cell count, using a Bürker chamber, and adjusted to the desired concentration.

### 2.2. Growth Determination

*C. auris* and *C. albicans* yeast cells in log-phase were counted, resuspended at 1 × 10^7^ CFU/mL in 5 mL of YPD and then serially diluted in phosphate-buffered saline (PBS, Sigma-Aldrich, Milan, Italy). Twenty μL of each yeast suspension were spotted in a plate of SDA + CAF. The plates were then incubated at 25 °C, 37 °C and 42 °C for 24–48 h. After incubation, the spot growth was visually assessed.

### 2.3. Antifungal Susceptibility Testing of C. auris CDC B11903

Antifungal susceptibility testing (AFST) was performed using the Thermo Scientific™ SENSITITRE™ YEASTONE™ panels (Waltham, MA, USA) according to manufacturer’s instructions with minor modifications. Briefly, the *C. auris* CDC B11903 strain, grown on SDA at 30 °C, was sub-cultured at the same condition to ensure purity and viability. To prepare the inoculum, four single colonies of at least 1 mm in diameter were picked, suspended in sterile 0.85% sodium chloride solution, vortexed and adjusted using a Sensititre™ nephelometer to a transmittance equal to a 0.5 McFarland standard at a wavelength of 530 nm as a stock solution. Further, 20 μL of the stock solution were transferred to the Sensititre™ YeastOne™ (SYO) broth medium, and the final density of the working solution was adjusted to 2 × 10^3^ CFU/mL by a Bürker chamber, according to Clinical and Laboratory Standards Institute (CLSI) M27M44S guidelines [31]. Then, 100 µL of the stock solution were transferred into each well of the SYO panels and incubated without agitation at 25 °C, 37 °C and 42 °C for 24 h before visual reading. Evident yeast growth was observed as the colour changed from blue (negative, indicating no growth) to pink (positive, indicating growth). We assessed various antifungal agents, including fluconazole, voriconazole, itraconazole, isavucozonale, posaconazole, caspofungin, micafungin, anidulafungin and amphotericin B. Resistance was determined through tentative breakpoints provided by the CDC [32] for amphotericin B (≥2 μg/mL), caspofungin (≥2 μg/mL), micafungin (≥4 μg/mL) anidulafungin (≥4 μg/mL) and fluconazole (≥32 μg/mL) while no tentative minimal inhibitory concentration (MIC) breakpoints are available for other azoles. MIC were visually determined as the lowest concentration that produced visual inhibition, compared to the control growth. The results were expressed as geometric means (GM) of two independent experiments performed in duplicate.

### 2.4. Melanin Production

To analyse the melanization capacity of *C. auris*, we used a protocol described by Smith D.F.Q. and collaborators [16]. Briefly, *C. auris* cells (1 × 10^7^ CFU/200 μL/well in triplicate) were cultured at 25 °C, 37 °C and 42 °C for 9 days in a minimal medium containing 15 mM C_6_H_12_O_6_, 10 mM MgSO_4_, 29.4 mM KH_2_PO_4_, 13 mM Glycine, 3M Vitamin B1, 1 mM L-DOPA, at pH 5.5. On days 0, 6 and 9 the plates were visually assessed and photographed. *C. albicans* grown in the same experimental conditions and in medium alone served as internal controls. The experimental protocol used to test melanization, as well as results from *C. albicans*, were shown in Supplementary Figure S1A. To quantify signal intensity within the wells, images were analyzed using ImageJ2 software (version 2.14.0/1.54f). Each image was first converted to 8-bit grayscale (Image → Type → 8-bit). A region of interest (ROI) corresponding to the inner area of a well was manually defined. This same ROI was then applied to three different wells within each image to measure the mean gray value (Analyze → Measure), ensuring consistency in the area analyzed. The mean gray value represents the average pixel intensity within the selected region and was used as a quantitative measurement of signal intensity.

### 2.5. Determination of Haemolytic Activity

The haemolytic activity of *C. auris* was assessed using the method described by Luo et al. [33]. *C. albicans* was used as a positive control due to its significant haemolytic activity [33]. Briefly, yeasts strains were cultured on SDA at 37 °C for 24 h, then harvested, washed with sterile PBS, and adjusted to 1 × 10^8^ cells/mL using a haemocytometer. A 10-μL aliquot was spotted onto SDA supplemented with 3% glucose and 7% fresh sheep blood, followed by incubation at 25 °C, 37 °C and 42 °C in 5% CO_2_ for 48 h. Haemolysis was assessed by observing the formation of a translucent halo around the inoculum under transmitted light (iBright Imaging System, Thermo Fisher Scientific, Waltham, MA, USA). The diameters of the lysis zones and colonies were measured using a computerized image analysis system (iBright Analysis Software, version 5.3.0, Thermo Fisher Scientific, Waltham, MA, USA). Haemolytic activity (Hz) was determined following the method of Price et al. [34], calculating the ratio of the colony diameter (mm) to the total diameter of the colony plus the halo of the haemolysis zone. Assays were conducted in triplicate, and results were expressed as the mean ±  SD of three independent experiments. According to the Hz index, haemolytic activity was classified as follows: Hz = 1.0: no haemolytic activity; Hz = 0.999–0.700: low haemolytic activity; Hz = 0.699–0.400: moderate haemolytic activity; Hz = 0.399–0.100: high haemolytic activity.

### 2.6. Phospholipase Activity

Extracellular phospholipase activity was evaluated by the egg yolk agar plate method described by Price et al. [34]. Briefly, 10 μL of *C. auris* suspension (1 × 10^8^/mL) were spotted onto the egg yolk agar medium (Thermo Fisher Scientific, USA) and incubated at 25 °C, 37 °C and 42 °C for 4 days. After incubation, the diameter of precipitation zone around the colony was determined. *C. albicans* strain B90028 was used as a control strain. Phospholipase activity (Pz index) was calculated by applying the following formula:

$$Pz=\frac{Colony\;diameter}{Colony\;diameter+Zone\;of\;precipitation}$$

On the basis of Pz value, phospholipase activity was classified in 5 types as follows:

Pz value = 1: no phospholipase activity; Pz value = 0.90–0.99: weak phospholipase activity (+); Pz value = 0.80–0.89: poor phospholipase activity (++); Pz value = 0.70–0.79: moderate phospholipase activity (+++); Pz value < 0.70: intense phospholipase activity (++++).

### 2.7. Lipase Activity

Lipase activity of *C. auris* and *C. albicans* was performed as previously described, with minor modifications [35]. Briefly, 20 μL of *C. auris* and *C. albicans* suspensions (1 × 10^8^/mL) were spotted onto an agar medium prepared as follows: 10.0 g of Bacto Peptone (BD Biosciences, Franklin Lakes, NJ, USA), 5.0 g of NaCl, 0.1 g of CaCl_2_, 15.0 g of agar in 1000 mL of distilled water. After sterilization by autoclave, the medium was allowed to cool to about 50 °C and 5 mL of Tween 80 (Sigma-Aldrich) were added. The plates inoculated with yeasts were incubated at 25 °C, 37 °C and 42 °C for 48 h. After incubation, the production of lipase (Lz) was expressed as the ratio of diameter of a colony to the total diameter plus zone of precipitation. The results were expressed as the average of the values obtained. The ranges of activity according to the Lz index were established as follows: high: Lz = 1: none; Lz value = 0.90–0.99: weak; Lz value = 0.70–0.89: moderate; Lz value ≤ 0.69: high [36].

### 2.8. Germ Tube Test

The ability of *C. auris* to germinate was assessed using the germ tube test [37]. *C. albicans* was used as a positive control. Briefly, the strains were grown on SDA + CAF for 24 h at 30 °C. After incubation, three different isolated *C. auris* and *C. albicans* colonies of at least 1 mm diameter were inoculated into 0.5 mL of FCS and incubated at 25 °C, 37 °C and 42 °C, respectively. After 3 h of incubation, 10 μL of *Candida* cell suspension in FCS were transferred onto a glass slide for examination by a light microscope (Olympus, Carl Zeiss, Oberkochen, Baden-Württemberg, Germany) with 100× magnification objective lenses. Images from one representative experiment carried out at each temperature were captured.

### 2.9. Filamentous Growth Assay

To evaluate the filamentous growth in *C. auris*, 2 × 10^3^ yeast cells were cultured in 96-well plates in 200 μL of RPMI 1640 medium, supplemented with 10% FCS, and incubated at 25 °C, 37 °C and 42 °C for 6 h and 24 h. *C. albicans* was used as a positive control. After incubation, the non-adherent cells were removed by washing the wells three times with PBS and the filamentous growth was visualized microscopically by a light microscope (Olympus, Carl Zeiss) with 40× magnification objective lenses. The images were documented with the accompanied digital camera.

### 2.10. Biofilm Quantification by Crystal Violet and XTT Assays

To evaluate the in vitro biofilm formation by *C. auris* and *C. albicans* at different temperatures, 2 × 10^5^ yeast cells were cultured in 96-well plates in 200 μL of RPMI 1640 medium (pH 7.0), supplemented with 10% FCS and incubated at 25 °C, 37 °C and 42 °C for 24 h. After incubation, planktonic *Candida* cells were gently aspirated, and the wells were washed at least thrice with warm PBS. The biofilm biomass and metabolic activity were evaluated by using crystal violet (CV) staining and 2,3-Bis-(2-Methoxy-4-Nitro-5-Sulfophenyl)-2H-Tetrazolium-5-Carboxanilide (XTT) reduction assay, respectively, as previously described [38]. In all experiments, the absorbance values of the negative control wells (containing no yeast cells) were subtracted from the values of the test wells to account for any background absorbance.

### 2.11. Human Epithelial Cells and Infection Protocol

In the experiments involving epithelial cell infection, two different human cell lines were employed. The urothelial cell line, T24 cells (Cytion 300352), were cultivated in Dulbecco Modified Eagle Medium/Nutrient Mixture F-12 (DMEM-F12) (Sial, Roma, Italy) supplemented with L-glutamine (2 mM), penicillin (100 U/mL) (Euroclone SpA, Pero, Italy), streptomycin (100 μL/mL) (Euroclone SpA, Italy) and 10% Fetal Bovine Serum (FBS, Sial, Italy), and incubated at 37 °C + 5% CO_2_. The pulmonary cell line, BEAS-2B cells (ATCC CRL-3588) were cultivated in DMEM High Glucose (Sial, Italy) supplemented with L-glutamine (2 mM, Sial, Italy), penicillin (100 U/mL) (Euroclone SpA, Italy), streptomycin (100 μL/mL) (Euroclone SpA, Italy), and 10% Fetal Bovine Serum (FBS, Sial, Italy), and incubated at 37 °C + 5% CO_2_. Both cell lines were kept viable by subculturing twice a week.

For generating epithelial monolayer, 5 × 10^5^ cells were seeded into the wells of a 24-well plate (SPL Life Sciences, Pocheon-si, Republic of Korea) (1 mL/well) and incubated at 37 °C + of 5% CO_2_ (48 h for T24 cells, 24 h for BEAS-2B cells). Before being infected, epithelial monolayers were washed with Phosphate-Buffered Saline (PBS, Sial, Italy), and fresh medium supplemented with 5% FBS was added to each well. Cells were infected with 5 × 10^5^ colony forming units (CFU) of yeasts (Multiplicity of Infection, MOI, 1) obtained from a YPD broth yeast culture grown overnight at 37 °C under agitation. After the infection, the plate was incubated at 37 °C + of 5% CO_2_.

### 2.12. Adhesion onto Abiotic and Biotic Substrates

The *C. auris* and *C. albicans* adhesion to abiotic and biotic surfaces was evaluated at different temperatures. To assess the adhesion on abiotic surface, 1 mL of fungal suspension (5 × 10^5^ CFU/mL) in YPD broth (Condalab, Madrid, Spain) was seeded into the wells of a 24-well plate in triplicate. The plate was incubated at 25 °C, 37 °C, or 42 °C for 90 min. Subsequently, the medium was removed, and the wells were washed with PBS to remove non-adherent yeasts. Yeast cells adhered to the plastic were detached by adding Soybean Casein Digest Lecithin Polysorbate 80 Medium (SCDLP-80, Biotec, Dueville, Italy) and vigorously pipetting. Samples were diluted, plated onto Sabouraud Dextrose Agar (SDA, Oxoid, Basingstoke, UK) and incubated at 37 °C for 24/48 h. Colony Forming Units (CFU) were counted, and the percentage of adhesion was calculated considering the inoculum as 100%.

T24 or BEAS-2B epithelial cell monolayer was infected with 5 × 10^5^ CFU (MOI 1) of *C. auris* or *C. albicans* and incubated at 37 °C or 42 °C for 90 min. Then, the medium was removed, and non-adherent yeast cells were removed through a PBS wash. Epithelial cells were lysed by adding 1 mL of 0.2% Triton X-100 (Sigma Aldrich, USA) to the wells and pipetting. Samples were diluted, plated onto SDA, and incubated at 37 °C for 24–48 h. Colony Forming Units (CFU) were counted, and the percentage of adhesion was calculated as above.

### 2.13. Statistical Analysis

The Shapiro–Wilk test was used to analyse the distribution of data within experimental groups. All statistical analyses were performed by using GraphPad Prism 10.3 software.

Statistical differences between groups were assessed by One-Way ANOVA followed by Tukey’s multiple-comparisons test or by Two-Way ANOVA followed by Sidak’s multiple-comparison test.

Values of * *p* < 0.05, ** *p* < 0.01, *** *p* < 0.001 and **** *p*< 0.0001 were considered statistically significant.

## 3. Results

### 3.1. Impact of Temperature on C. auris Growth

**Figure 1:**
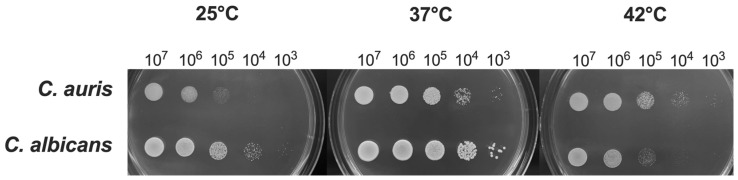
**Impact of temperature on** ***C. auris*** **and** ***C. albicans*** **growth.** *C. auris* and *C. albicans* yeasts cells (20 μL from 1 × 10^7^ CFU/mL to 1 × 10^3^ CFU/mL) were spotted on SDA + CAF and allowed to grow at 25 °C, 37 °C and 42 °C for 24 h. A representative image out of 4 with similar results is shown.

Previous studies reported that the optimal range of temperatures for *C. auris* is between 37 °C and 40 °C, similarly to *C. albicans* [2]. However, some *C. auris* isolates have been shown to grow at temperatures as high as 42 °C, a condition that inhibits the growth of *C. albicans* [39]. In the present study, we evaluated the impact of different temperatures on the growth of *C. auris* reference strain CDC B11903 and we compared it to the growth of *C. albicans* B90028 under the same experimental conditions. The temperatures were selected to mimic real conditions, i.e., temperatures easy to find in nature (25 °C) and anthropic environments, including normal human body temperature (37 °C), and the temperature occurring in patients with severe febrile status (42 °C) in response to an infectious disease. By spotting *C. auris* on SDA + CAF at different concentrations, we could not detect any visible difference in fungal growth for the concentration of 1 × 10^7^ CFU/mL, irrespective of the growing temperature. However, by reducing the fungal inoculum we observed a growth impairment at 25 °C (and to a lesser extent at 42 °C), as compared to the optimal growing temperature of 37 °C. Notably, *C. auris* growth was impaired at 25 °C starting from the concentration of 1 × 10^5^ CFU/mL while *C. auris* growth seemed to be almost unchanged at 42 °C even at low inoculum (i.e., down to 1 × 10^3^ CFU/mL). Conversely, as expected, *C. albicans* showed to be most hindered in growing at 42 °C (Figure 1).

### 3.2. Impact of Temperature on C. auris MIC

First, we evaluated the antifungal susceptibility of *C. auris* at 25 °C, 37 °C and 42 °C. The data shown in Table 1 indicate that the strain was sensitive to all the tested antifungal agents. Moreover, the results highlight the influence of temperature on the antifungal susceptibility profile of *C. auris*. In fact, the susceptibility to all the antifungals tested was significantly reduced from 25 °C to 37 °C and it remained stable at temperatures ranging from 37 °C to 42 °C (from 0.06 to 0.12 μg/mL), except for anidulafungin, for which no differences were observed in MIC values at temperatures between 25 °C and 37 °C.

### 3.3. Impact of Temperature on C. auris Melanization

**Figure 2:**
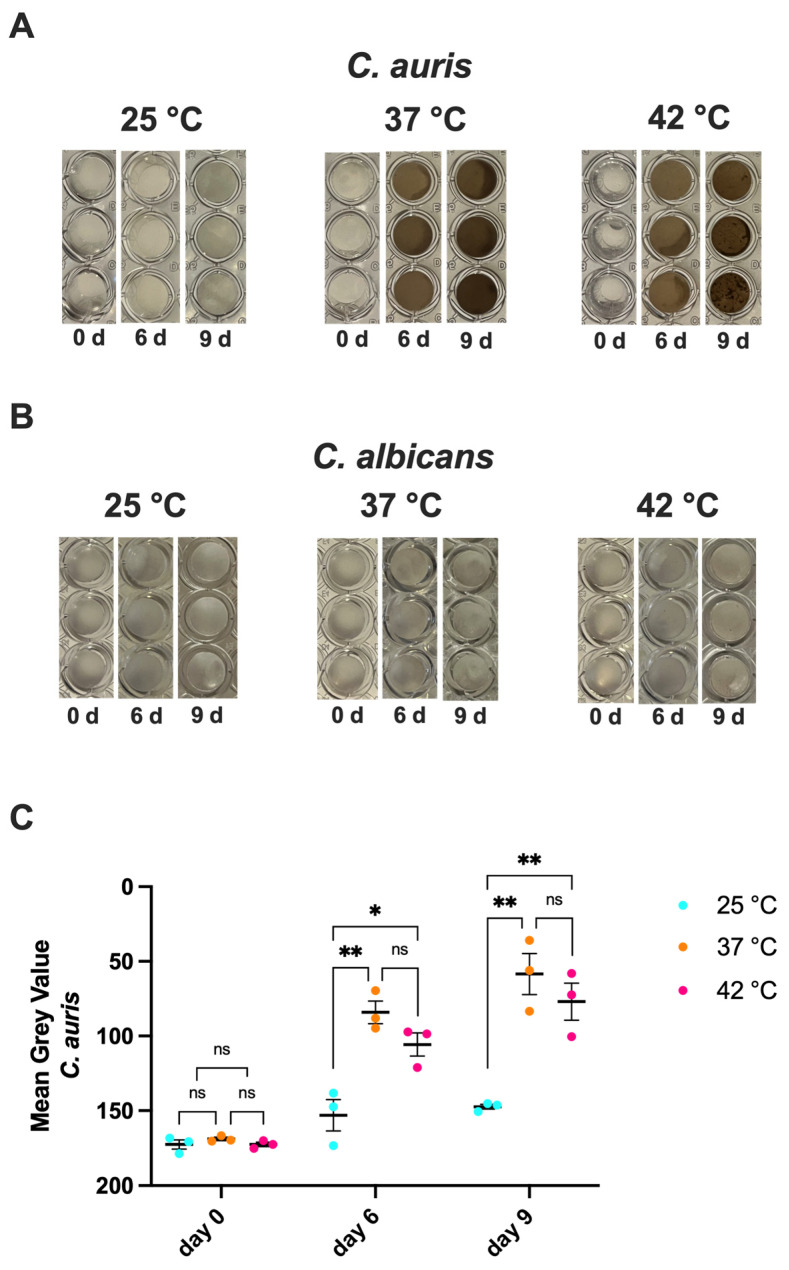
**Impact of temperature on** ***C. auris*** **and** ***C. albicans*** **melanin production.** *C. auris* CDC B11903 melanization capacity after 0, 6 and 9 days at 25 °C, 37 °C and 42 °C. A representative image out of 3 with similar results is shown (**A**). A representative image of *C. albicans* B90028 melanization capacity after 0, 6 and 9 days at 25 °C, 37 °C and 42 °C is shown (**B**). Mean grey value ± SEM of melanin production by *C. auris* CDC B11903 (**C**). Differences between groups were analyzed by One-Way ANOVA followed by Tukey’s multiple comparisons test. * *p* < 0.05; ** *p* < 0.01; ns, not significant.

Melanin is a black-brown pigment typically produced following enzymatic oxidation of aromatic precursors and it promotes fungal virulence by different mechanisms. Literature reports that several *C. auris* strains are able to produce melanin [16]. To evaluate this capacity in *C. auris* CDC B11903, the fungus was cultured in a specific medium containing melanin precursor, at 25 °C, 37 °C and 42 °C. The results in Figure 2A,B show that *C. auris* was able to produce melanin after 6 and 9 days only at 37 °C and at 42 °C, with no significant differences between these two temperatures. No melanin production was observed at 25 °C. Moreover, as expected, no melanin production was observed for *C. albicans*, used as a negative control, under the same experimental conditions (Figure 2B).

### 3.4. Impact of Temperature on C. auris Haemolytic Activity

**Figure 3:**
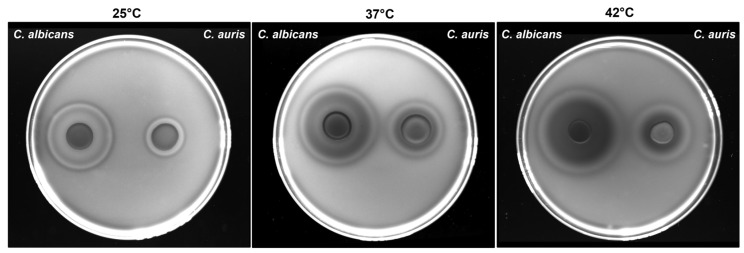
**Impact of temperature on** ***C. albicans*** **and** ***C. auris*** **haemolytic activity.** *C. auris* CDC B11903 and *C. albicans* B90028 haemolytic activity at 25 °C, 37 °C and 42 °C. A representative image out of 3 with similar results is shown.

As shown in Figure 3, *C. auris* exhibited haemolytic activity at all three tested temperatures with significantly larger halos observed at 37 °C and 42 °C, as compared to 25 °C. Notably, two types of haemolysis were observed in both yeasts: a translucent ring indicative of β-haemolysis, detected at all three temperatures, and a greenish-black halo suggestive of α-haemolysis, observed only at 37 °C and 42 °C. To quantify haemolytic activity, the haemolytic index (Hz) was calculated as the ratio between the diameter of the colony and the total diameter of the colony plus halo. As shown in Table 2, *C. auris* exhibited low haemolytic activity at 25 °C, moderate at 37 °C and high at 42 °C. As expected, *C. albicans* demonstrated high haemolytic activity at all the tested temperatures.

### 3.5. Impact of Temperature on C. auris Phospholipase and Lipase Activity

**Figure 4:**
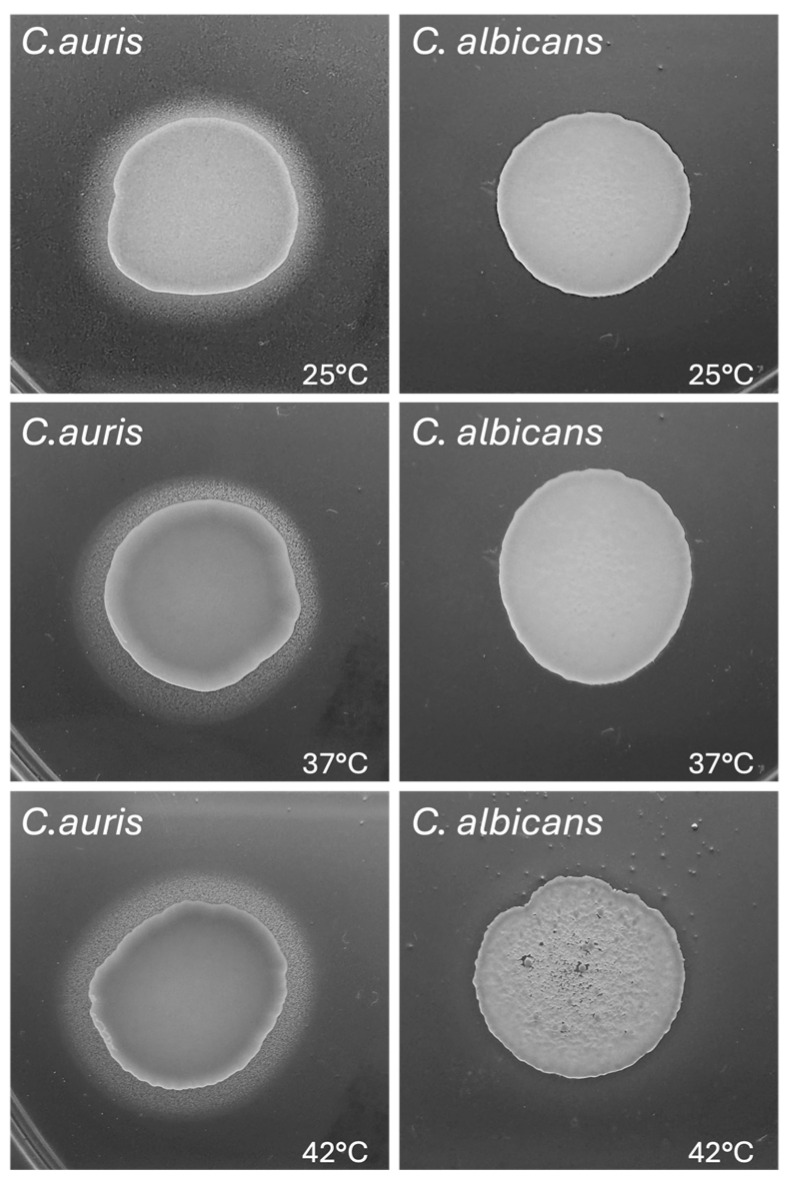
**Impact of temperature on** ***C. auris*** **and** ***C. albicans*** **phospholipase activity.** *C. auris* CDC B11903 and *C. albicans* B90028 phospholipase activity at 25 °C, 37 °C and 42 °C. A representative image out of 3 with similar results is shown.

Phospholipase activity plays an important role in *Candida* pathogenicity [40]. In the present work we evaluated the impact of temperature on extracellular phospholipase activity of *C. auris* CDC B11903 in comparison to *C. albicans* B90028. As reported in Figure 4 and in Table 3, *C. auris* displayed a moderate phospholipase activity at all three tested temperatures, as suggested by the Pz values of 0.78 at 25 °C and 37 °C and 0.77 at 42 °C. Differently, *C. albicans* did not show any phospholipase activity irrespective of the temperature tested.

**Figure 5:**
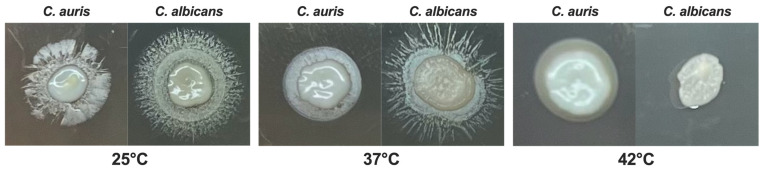
**Impact of temperature on** ***C. auris*** **and** ***C. albicans*** **lipase activity.** *C. auris* CDC B11903 and *C. albicans* B90028 lipase activity at 25 °C, 37 °C and 42 °C after 48 h of incubation. One representative image out of three with similar results is shown.

Concerning the lipase activity, our results show that, after 48 h of culture, both *C. auris* and *C. albicans* exert high lipase activity at 25 °C. The lipase activity was maintained at 37 °C both for *C. auris* (moderate activity) and *C. albicans* (high activity). Conversely, while *C. auris* showed lipase production also at 42 °C (moderate activity), *C. albicans* failed to produce lipase at 42 °C (Figure 5 and Table 4).

### 3.6. Impact of Temperature on C. auris Germ Tube Formation

**Figure 6:**
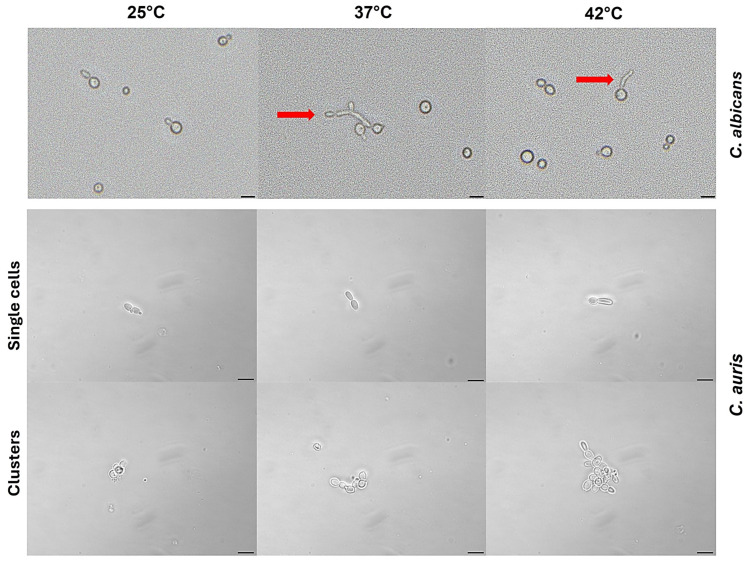
**Effect of temperatures on germ tube formation in** ***C. albicans*** **and** ***C. auris*****.** Yeasts cells were cultured in the presence of 100% FCS at 25 °C, 37 °C and 42 °C. Germ tube formation was analysed by light microscopy after 3 h of incubation. *C. albicans* was used as a positive control, showing positive germ tube test (red arrows). Images were acquired by a light microscope with 40× magnification objective lenses. One representative experiment is shown for each temperature condition. Scale bar = 10 µm.

The morphological transition of *C. albicans* from yeast to filamentous form is influenced by several laboratory conditions, including temperature of 37 °C and the presence of serum, both mimicking the host tissue environment occurring in mammals [41]. Here, we evaluated the ability of *C. auris* strain CDC B11903 to germinate in the presence of 100% FCS under the temperatures of 25 °C, 37 °C and 42 °C. The germination was assessed by evaluating both single cells and clusters. As shown in Figure 6, *C. auris* failed to produce germ tubes under the above-mentioned conditions, in contrast to *C. albicans*, used as an internal control, which exhibited a positive germ tube test at 37 °C and 42 °C (Figure 6), with subsequent development into true hyphae and pseudohyphae.

### 3.7. Impact of Temperature on C. auris Pseudohyphal Formation

**Figure 7:**
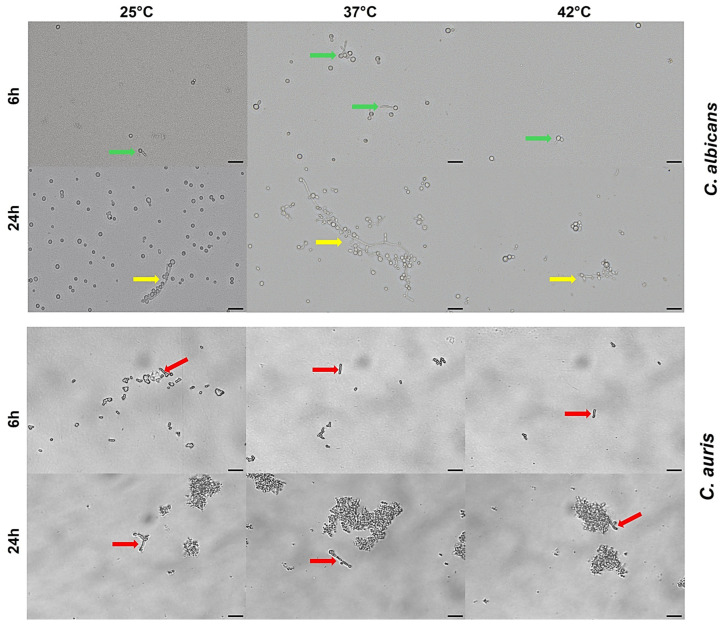
**Effect of temperature on yeast-to-pseudohyphae phenotypic switching** **in** ***C. albicans*** **and** ***C. auris*****.** Yeasts cells were cultured in RPMI 1640 supplemented with 10% FCS at 25 °C, 37 °C and 42 °C for 24 h. The phenotypic transition from yeast-to-pseudo-hyphal/hyphal morphotype was analysed by light microscopy at 6 h and 24 h. At all tested temperatures, pseudo-hyphal growth was observed only in single *C. auris* cells (red arrows), but not in clustered cells. *C. albicans* was used as an internal control, showing phenotypic switching from yeast to filamentous form as pseudohyphae (green arrows) or true hyphae (yellow arrows). Images were acquired by a light microscope with 40× magnification objective lenses. One representative experiment out of three for each temperature is shown. Scale bar: 20 µm.

The phenotypic switching from yeast to filamentous form as true hyphae or pseudohyphae is a key virulence factor in *C. albicans* and it is influenced by environmental conditions, both in vitro and in vivo. Unlike *C. albicans*, the pseudo-hyphal is the only filamentous morphology assumed by *C. auris* in in vitro experimental models, even under genotoxic stress conditions, while parallel-sided true hyphae have never been observed [42]. In addition, the capability of *C. auris* to switch from yeast to pseudo-hyphal filamentous form is strain specific, thus suggesting the heterogenous nature of this species [41]. In the present study we evaluated the impact of different temperatures (25 °C, 37 °C and 42 °C) on the capability of *C. auris* CDC B11903 to grow as pseudo-hyphal form. The microscope images reported in Figure 7 show that, under our experimental conditions, the temperature did not influence the capability of *C. auris* to form pseudo-hyphae. Indeed, the filamentous growth was quite similar after 6 and 24 h exposure to all temperatures tested and more evident in *C. auris* single yeasts than in clustered cells. Moreover, the images also show the ability of *C. auris* CDC B11903 to coaggregate after 24 h of culture under the above-mentioned experimental conditions at all the temperatures tested. *C. albicans* was used as an internal control, showing phenotypic switching from yeast to filamentous form as true hyphae or pseudohyphae (Figure 7).

### 3.8. Impact of Temperature on C. auris Biofilm Formation and Metabolic Activity

*C. auris* CDC B11903 biofilm production was evaluated in terms of total biomass by CV staining and metabolic activity using the colorimetric XTT reduction assay. The data show that there were no statistically significant differences in *C. auris* biofilm biomass irrespective of the temperature (Table 5). Contrary, the metabolic activity of the biofilm has shown to be temperature-dependent, as suggested by the higher OD values reached at 37 °C, compared to those observed at 42 °C and 25 °C (* *p* < 0.05 and ** *p* < 0.01, respectively) (Table 5) *C. albicans*, used as a positive control, showed a significant increase in biofilm biomass at 37 °C and 42 °C compared to 25 °C (** *p* < 0.01), along with a moderate but significant increase in metabolic activity (* *p* < 0.05) (Table 5).

### 3.9. Adhesion Capacity of C. auris to Abiotic Surfaces and Epithelial Cells at Different Temperatures

**Figure 8:**
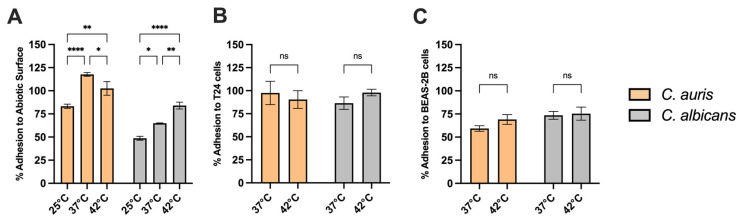
***C. auris*** **and** ***C. albicans*** **adhesion to abiotic surfaces and epithelial cells.** The impact of the different temperatures on adhesion of *C. auris* CDC B11903 and *C. albicans* 90028 was evaluated on abiotic surfaces (**A**), T24 urothelial cells (**B**) and BEAS-2B bronchial epithelial cells (**C**). Data are the mean ± SEM of three independent experiments performed in triplicate. Differences between groups were analysed by Two-Way ANOVA followed by Sidak’s multiple-comparison test. ns, not significative; * *p* < 0.05; ** *p* < 0.01; **** *p* < 0.0001.

We next examined how temperature variation impacts on *C. auris* adhesion to different substrates: abiotic surfaces and epithelial cells such as the human bronchial epithelial cell line BEAS-2B and the T24 urothelial cell lines. Our results show that *C. auris* exhibited an increased capacity to adhere to abiotic surfaces at all the temperature tested, compared to *C. albicans*. Notably, its adhesion capacity increased with rising temperature, from 25 °C to 37 °C, and remained constant up to 42 °C (Figure 8A). The temperature did not affect *C. auris* adhesion to epithelial cells, such as T24 urothelial cells (Figure 8B) and BEAS-2B cells (Figure 8C) Indeed, no statistically significant differences were observed in *C. auris* adhesion between 37 °C and 42 °C. Furthermore, no significant differences were observed between *C. auris* and *C. albicans* adhesion to epithelial cells at both temperatures.

## 4. Discussion

Survival and growth at physiological body temperature in humans are essential prerequisites for microbial invasion and pathogenicity. Most fungal pathogens are adapted to environmental conditions and thus they do not grow well at relatively high temperatures, as those occurring within the human body, both in physiological conditions (37 °C) or in severe febrile status (over 40 °C) [43,44]. Interestingly, *C. auris* strains differ in their growth and virulence factors in vitro. The ability of some *C. auris* isolates to grow at 37 °C and 40 °C appears to be similar to that of *C. albicans*, and certain isolates are able to grow even at 42 °C [45]. In this study we have evaluated the impact of different temperatures on biological properties and antifungal susceptibility of *C. auris* reference strain CDC B11903. This strain has been found to grow better at temperatures ranging from 37 °C to 42 °C rather than at 25 °C. Moreover, no difference in biofilm formation has been observed at any of the temperatures tested in terms of total biomass production, however, a reduction in biofilm metabolic activity has been observed both at 25 °C and at 42 °C, with respect to 37 °C. The 25 °C temperature has shown a negative impact on *C. auris* growth, when the fungal inoculum had been reduced. This is in line with the data obtained by the antifungal susceptibility test, which show lower values of MIC at 25 °C as compared to 37 °C, while no difference in MIC values has been observed between 37 °C and 42 °C (except for anidulafungin, whose MIC value has increased at 42 °C). These data suggest that in our experimental condition *C. auris* CDC B11903 has the capacity to tolerate high temperatures when exposed to drastic increases. On the other hand, temperatures lower than 37 °C seem to have a more negative impact on the fungus, since no growth has been observed at inoculum below 1 × 10^5^ CFU/mL and the MIC values are lower.

A growing number of emerging cases of *C. auris* have been reported with fungal resistance to the standard antifungal treatments including azoles, echinocandins, and polyenes, making these infections difficult to treat [46]. In the United States, about 90% of *C. auris* isolates have been reported to be resistant to fluconazole, about 30% to amphotericin B, and less than 2% to echinocandins [46,47]. Fungal resistance is one of the paramount points in antifungal stewardship. It has been demonstrated that *C. albicans* is more susceptible to caspofungin at 37 °C than at 30 °C, in a calcineurin-dependent pathway and not related to *FKS*, *CHS*, or *CHT* genes, all involved in echinocandins resistance [48]. Based on this evidence, we have performed AFST at the three different temperatures of 25 °C, 37 °C and 42 °C for *C. auris* CDC B11903. First, the Sensititre™ YeastOne™ YO10 AST Plate (Thermofisher, Milan, Italy) investigation, with our minor modifications, has worked at all the tested temperatures, supporting the employment of this method for *C. auris* AFST profile in clinical and research settings, as previously reported [49]. Regarding the obtained MIC, our results show that the strain CDC B11903 does not exhibit any resistance to the tested drugs. Indeed, variations in MIC have been highlighted at the selected temperatures, with the lowest values observed at 25 °C for all the tested antifungal drugs and increasing values at 37 °C and 42 °C. Interestingly, these results are in contrast to what had been reported for *C. albicans* [48]. Recent pharmacokinetic/pharmacodynamic analysis of *C. auris* in a mouse model of infection indicates that, under standard dosing, the breakpoint for amphotericin B should be 1 or 1.5, i.e., values similar to those determined for other *Candida* species [50]. Therefore, isolates with a MIC ≥ 2 should now be considered resistant. In our case, *C. auris* exhibits an AmB MIC of 0.5 at 25 °C and at 37 °C, while the high temperature of 42 °C results to have increased the MIC value to 1 µg/mL. The temperature-dependent increase in AmB MIC for *C. auris* suggests a potential reduction in efficacy under febrile conditions, thereby challenging the reliability of current breakpoints, particularly for clinical isolates of *C. auris* and in regions where AmB resistance or elevated MICs have been reported. This underscores the need for susceptibility testing that better reflects clinical realities, especially in immunocompromised patients. Antifungal tolerance is often associated with metabolic dormancy, a reversible low-activity state that reduces drugs efficacy [51,52,53]. Thus, lower MIC at 25 °C may reflect a decrease in metabolic activity and a downregulation of targets like 14-demethylase, β-(1,3)-D-glucan synthase, and ergosterol biosynthesis enzymes [52]. Indeed, it has been suggested that higher temperatures accelerate fungal growth and increase susceptibility to drugs targeting metabolism and cell division (e.g., fluconazole, ketoconazole, 5-fluorouracil) [54]. Similarly, it has been reported that elevated temperatures enhance lipid synthesis, potentially sensitizing fungi to amphotericin B and sterol-inhibiting azoles [55]. In addition, higher temperatures promote azole uptake through increased membrane solubility [56,57]. Differently, our data show that while metabolic activity peaked at 37 °C, MIC remained stable compared to 42 °C, suggesting that thermally induced tolerance mechanisms, rather than metabolic output alone, may drive resistance phenotypes. Although in this study we focused on *C. auris* antimicrobial profile in response to rising temperature, future investigations will explore the transcriptional regulation of *C. auris* antifungal susceptibility in response to different temperatures.

The capacity of *C. auris* strain CDC B11903 to tolerate high temperatures is only one of the characteristics that delineates its capacity to behave as an opportunistic pathogen. Several non-pathogenic fungi can grow at temperatures around 37 °C. Indeed, all the fungal species belonging to the mycobiome are tolerant to the human body temperature and only a few of them cause disease and mostly in severely immunocompromised subjects [58]. It follows that this prerequisite must be owned also by those fungi that have adapted to grow within the human body. Hence, the thermotolerance displayed by *C. auris* strain CDC B11903 is not sufficient, *per se*, to define it as a potentially opportunistic fungal pathogen [59]. It is acknowledged that what keeps harmless the members of the commensal mycobiota, in comparison to opportunistic fungal pathogens, is not the capacity to grow at body temperature, but rather the lack of additional virulence factors. Therefore, we analysed the presence of virulence traits of *C. auris* CDC B11903 strain that are expressed at body temperature. The production of melanin is an important virulence trait of several *C. auris* strains [16]. Here, we show for the first time that *C. auris* strain B11903 can produce melanin when grown at 37 °C and 42 °C, further suggesting its adaptation to the human body temperature: indeed, in such condition this strain can survive, grow and express its virulence factors. Fungal melanin pigments have strong antioxidant properties that allow them to resist oxidative damage caused by the host immune cells, such as macrophages and neutrophils oxidative bursts [60]. In addition, melanin can bind to and inactivate antimicrobial peptides and antimicrobial enzymes that the host typically uses to degrade and kill fungi during infection, as well as antifungal drugs used to treat infections [61,62,63]. Fungal melanin, located in the fungal cell wall, can alter cell wall composition and physically mask pathogen-associated molecular patterns (PAMPs) that would otherwise be recognized and bound by host’s pathogen recognition receptors (PRRs) [16]. These changes may lead to diminished recognition by host immune cells. Hence, our results point to a key role exerted by melanin at 37 °C and 42 °C that increases resistance to fungal drugs and immune responses.

One of the key virulence attributes of pathogenic fungi, including *Candida* species, is their ability to acquire nutrients and breach host barriers through the secretion of hydrolytic enzymes and toxins. In this study, we have assessed the production of haemolysins, phospholipases and lipases by *C. auris* CDC B11903 at different temperatures representative of environmental and host conditions. The haemolytic capacity of *C. auris* is evident at all the tested temperatures, though more pronounced at 37 °C and 42 °C with respect to 25 °C, as shown by the wider haemolytic halos. Both β-haemolysis and α-haemolysis have been detected, with the latter emerging exclusively at the higher temperatures. This dual haemolytic profile has been reported also in other clinical isolates of *C. auris*, particularly under host-mimicking conditions [64] and it may reflect an adaptive response to evade host immune defences through the lysis of red blood cells. However, unlike *C. albicans*, which displays strong haemolytic activity at all temperatures, the performance of *C. auris* at 25 °C remains low, suggesting that its full virulence potential may require physiological or febrile host temperatures. This observation supports the hypothesis that temperature acts as a signal for virulence activation, as previously demonstrated in *C. albicans* [65]. Further investigations are required to elucidate the nature of the haemolytic factor, specifically to determine whether it is enzymatic or toxin-mediated.

Phospholipase activity, a well-known virulence trait associated with host cell membrane disruption and tissue invasion, has been detected consistently in *C. auris* CDC B11903 across all the tested temperatures. The Pz values, which remain in a narrow range (0.77–0.78), indicate a moderate but stable phospholipase production. This thermostability suggests that *C. auris* maintains a constitutive phospholipase expression regardless of environmental conditions, including host-relevant febrile temperatures. Such stability may represent an adaptive advantage, particularly during colonization and systemic infection, where fluctuating temperatures could otherwise impair enzymatic virulence responses. Interestingly, previous studies have reported inconsistent phospholipase activity in *C. auris* clinical isolates, with some strains exhibiting little to no detectable enzymatic production [45,66]. Our findings, therefore, contribute to the growing evidence that phospholipase expression in *C. auris* is strain-dependent and not uniformly absent or negligible as initially suggested in early characterizations of this species. The persistence of phospholipase activity across physiological and stress temperatures in this strain supports its potential contribution to pathogenicity, particularly in niches where host membranes represent a primary barrier to fungal invasion.

Moreover, lipase activity appears to be influenced by temperature in *C. auris*, with a high and moderate lipase production at 25 °C and 37 °C, respectively, which is also maintained at 42 °C. These results align with previous findings indicating that lipase expression is regulated under host-mimicking conditions and may play a role in skin colonization and biofilm formation [66]. Conversely, *C. albicans* does not show lipase activity at 42 °C, confirming that the expression of this enzyme is highly variable and context-dependent, even among established pathogens. The lipase activity of *C. auris* also at febrile-range temperatures may confer this species a selective advantage during systemic infection, or in the colonization of warm, lipid-rich niches such as skin folds. Moreover, given the high lipophilicity of human skin and medical devices, this enzymatic activity may underlie the persistence and transmission of *C. auris* in healthcare settings. Nonetheless, our data highlight the importance of not underestimating enzymatic virulence factors in *C. auris*, which may play a more active role in pathogenesis than previously recognized.

Moreover, our results show that temperatures ranging from 25 °C to 42 °C, do not substantially affect pseudo-hyphae formation in *C. auris*. Global warming has been proposed as a contributing factor in the generation and dissemination of high thermotolerant *C. auris* strains [67,68,69]. In line with previous studies, the *C. auris* strain CDC B11903 has been unable to form true hyphae, when cultured in RPMI 1640 supplemented with 10% FCS, or in the presence of 100% FCS. Under these experimental conditions, only pseudo-hyphae, stemming from single yeast cells rather than from cells gathered in clusters have been detected. The production of pseudo-hyphae as a virulence factor in planktonic cells is supported also by previous in vivo experimental models showing that non-aggregating isolates of *C. auris* exhibit more pathogenicity than aggregating isolates; in addition, such non-aggregating isolates have been shown to be even more virulent than *C. albicans* [15]. On the other hand, strains capable of forming aggregates have been shown to be more resistant to disinfectants, azole antifungals and immune responses. In addition, they are characterized by a higher ability to colonise (and persist on) biotic and abiotic surfaces. Here, we show an aggregative profile for *C. auris* CDC B11903. Both pseudo-hyphae formation and aggregative capacity are not modified by changing the temperature from 25 °C to 42 °C.

Adherence to both abiotic and biotic surfaces constitute a pivotal early event in fungal pathogenesis, enabling colonization, biofilm development and persistence within host tissues and healthcare settings [70]. In this study, *C. auris* CDC B11903 exhibits significantly higher adhesion capacity to abiotic substrates than *C. albicans* throughout all the tested temperatures, with adhesive capacity increasing from 25 °C to 37 °C and stabilizing at 42 °C. This thermally responsive trend suggests optimal activation of adhesion-related mechanisms at human body temperature, with maintained performance under febrile conditions, reflecting the induction of adhesins or a cell wall restructuring. The enhanced adhesion under both normothermic and hyperthermic conditions may facilitate early colonization and biofilm establishment, complicating eradication efforts due to inherent antifungal tolerance.

Furthermore, *C. auris* adheres efficiently to T24 urothelial and BEAS-2B bronchial epithelial cells at both 37 °C and 42 °C, indicating a broad thermotolerance in host-surface interactions. This epithelial tropism may partially explain its frequent detection in urinary and respiratory sites, particularly among critically ill or immunocompromised individuals. Collectively, these observations challenge earlier assumptions regarding the limited virulence of *C. auris*, instead revealing a consistent and temperature-resilient adhesive phenotype that may act synergistically with enzymatic activity and biofilm formation to promote infection. Overall, these findings provide a comprehensive view of the virulence arsenal of *C. auris* CDC B11903 and reinforce the notion that temperature-dependent regulation of specific traits—such as enzyme secretion and melanin production—may critically shape the pathogenic behavior of this emerging fungal pathogen. Although this study focused on phenotypic and susceptibility profiling of *C. auris*, future investigations will explore the transcriptional regulation of key virulent genes in response to thermal shifts to elucidate the molecular underpinnings of *C. auris* pathogenicity.

## 5. Conclusions

Our study demonstrates that *C. auris* CDC B11903 expresses several virulence factors in a temperature-dependent manner. While it seems to be more susceptible to antifungal drugs and less pathogenic at 25 °C, when exposed at 37 °C and at 42 °C it is able to increase its virulence. Further studies are warranted to explore the genotype-phenotype correlation and peculiar proteomic signatures at different environmental conditions.

## Figures and Tables

**Table 1:** Antifungal susceptibility testing of *C. auris* CDC B11903 at 25 °C, 37 °C and 42 °C. The values are the geometric means of the MICs (µg/mL).

Temperature	25 °C	37 °C	42 °C
Amphotericin B	0.50 μg/mL	1.00 μg/mL	1.00 μg/mL
Fluconazole	4.00 μg/mL	8.00 μg/mL	8.00 μg/mL
Isavuconazole	0.008 μg/mL	0.12 μg/mL	0.12 μg/mL
Itraconazole	0.03 μg/mL	0.12 μg/mL	0.12 μg/mL
Posaconazole	0.015 μg/mL	0.06 μg/mL	0.06 μg/mL
Voriconazole	0.015 μg/mL	0.06 μg/mL	0.06 μg/mL
Micafungin	0.03 μg/mL	0.06 μg/mL	0.06 μg/mL
Anidulafungin	0.06 μg/mL	0.06 μg/mL	0.12 μg/mL
Caspofungin	0.06 μg/mL	0.12 μg/mL	0.12 μg/mL

**Table 2:** Haemolytic activity of *C. auris* CDC B11903 and *C. albicans* B90028 at 25 °C, 37 °C and 42 °C. Hz: haemolytic index. The results are expressed as the mean ± SD of three independent experiments. Differences between groups were analyzed by One-Way ANOVA followed by Tukey’s multiple comparisons test. **** *p* < 0.0001 (*C. auris* 25 °C vs. 37 °C), ^####^ *p* < 0.0001 (*C. auris* 25 °C vs. 42 °C).

Temperature	25 °C		37 °C		42 °C	
	Hz	Categorization	Hz	Categorization	Hz	Categorization
*C. auris*	0.690 ± 0.073	low	0.46 ± 0.020 ****	moderate	0.38 ± 0.003 ^####^	high
*C. albicans*	0.376 ± 0.011	high	0.322 ± 0.036	high	0.251 ± 0.011	high

**Table 3:** Extracellular phospholipase activity of *C. auris* CDC B11903 and *C. albicans* B90028 at 25 °C, 37 °C and 42 °C. Pz: phospholipase activity index. The results are the mean ± SD of three independent experiments carried out in duplicate. Differences between groups were analyzed by One-Way ANOVA followed by Tukey’s multiple comparisons test. ns (*C. auris* 25 °C vs. 37 °C), ns (*C. auris* 25 °C vs. 42 °C).

Temperature	25 °C		37 °C		42 °C	
	Pz	Categorization	Pz	Categorization	Pz	Categorization
*C. auris*	0. 780 ± 0.055	moderate	0.780 ± 0.063	moderate	0.770 ± 0.030	moderate
*C. albicans*	1	none	1	none	1	none

**Table 4:** Lipase activity of *C. auris* CDC B11903 and *C. albicans* B90028 at 25 °C, 37 °C and 42 °C. Lz: lipase activity index. The results are the mean ± SD of three different experiments. Differences between groups were analyzed by One-Way ANOVA followed by Tukey’s multiple comparisons test. ^###^ *p* < 0.001 (*C. auris* 25 °C vs. 37 °C and 25 °C vs. 42 °C), ^###^ *p* < 0.001 (*C. albicans* 25 °C vs. 42 °C and 37 °C vs. 42 °C).

Temperature	25 °C		37 °C		42 °C	
	Lz Score	Categorization	Lz Score	Categorization	Lz Score	Categorization
*C. auris*	0.48 ± 0.014	High	0.72 ± 0.059 ^###^	Moderate	0.71 ± 0.044 ^###^	Moderate
*C. albicans*	0.46 ± 0.043	High	0.50 ± 0.113	High	1.00 ^###^	none

**Table 5:** **Effect of temperature on** ***C. auris*** **and** ***C. albicans*** **biofilm biomass and metabolic activity.** The impact of the different temperatures was evaluated on biofilm biomass and metabolic activity in *C. auris*. *C. albicans* was used as a positive control. Each value represents the mean ± SD of three independent experiments carried out in triplicate. Statistical analysis was performed by using One-way ANOVA followed by Tukey’s multiple comparisons test. * *p* < 0.05; ** *p* < 0.01; ns: not significant.

	Temperature	Biofilm Biomass (OD 595 nm)	*p*-Value	Biofilm Metabolic Activity (OD 490 nm)	*p*-Value
*C. auris*	25 °C	0.764 ± 0.234	-	0.580 ± 0.112	-
	37 °C	0.772 ± 0.236	ns	0.731 ± 0.083	** vs. 25 °C (*p* < 0.01)
	42 °C	0.749 ± 0.248	ns	0.614 ± 0.087	* vs. 37 °C (*p* < 0.05)
*C. albicans*	25 °C	0.889 ± 0.128	-	0.865 ± 0.173	
	37 °C	1.021 ± 0.125	* vs 25 °C (*p* < 0.001)	0.962 ± 0.173	* vs. 25 °C (*p* < 0.05)
	42 °C	1.019 ± 0.126	* vs. 25 °C (*p* < 0.001)	0.932 ± 0.173	* vs. 25 °C (*p* < 0.05)

## Data Availability

The original contributions presented in the study are included in the article, further inquiries can be directed to the corresponding authors.

## Supplementary Materials

The following supporting information can be downloaded at: https://www.mdpi.com/article/10.3390/jof11090625/s1, Figure S1: Schematic representation of the experimental protocol used to test the impact of temperature on *C. auris* melanin production.
