# Supplementary material for: Biological Features, Antimicrobial Susceptibility and Phenotypic Characterization of Candidozyma auris CDC B11903 Grown at Different Temperatures
Source: J Fungi (Basel). 2025 Aug 26;11(9):625. doi: 10.3390/jof11090625 (PMC12470741; doi:10.3390/jof11090625)
Supplement: Supplementary file 1 [file jof-11-00625-s001.zip › jof-3789499-supplementary.pdf]

Supplemental Materials:

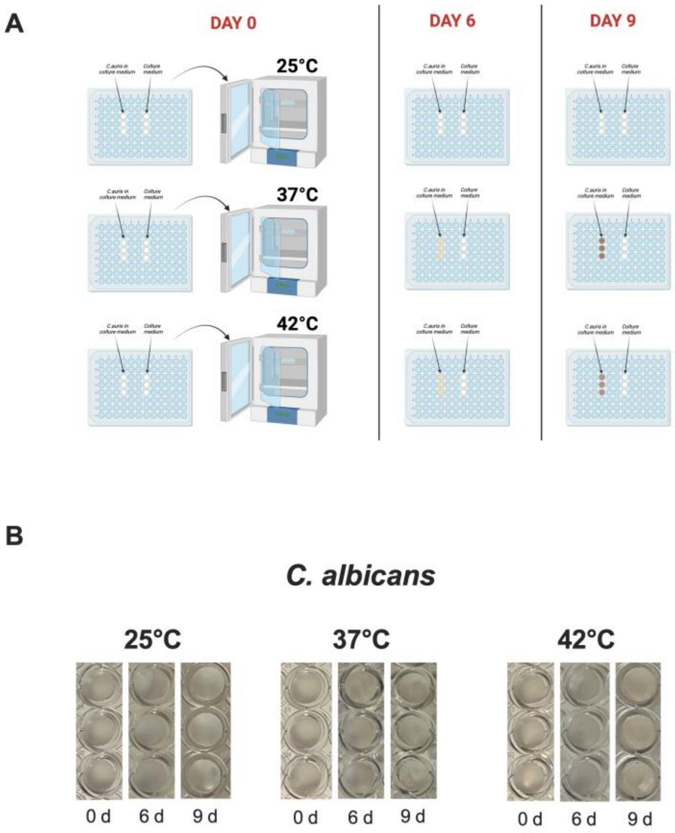

Supplementary Figure S1. (**A**) Schematic representation of the experimental protocol used to test the impact of temperature on Candida melanin production. **(B)** *C. albicans* B90028 melanization capacity after 0, 6 and 9 days at 25 °C, 37 °C and 42 °C. A representative image out of 3 with similar results is shown.
